# Predictive value of preoperative inflammatory response markers on short-term postoperative complications following colorectal surgery: a secondary analysis of a randomized clinical trial

**DOI:** 10.3389/fmed.2025.1536807

**Published:** 2025-06-05

**Authors:** Ning Bu, Chang Liu, Zhidong Kong, Wei Gao, Yaomin Zhu

**Affiliations:** ^1^Department of Anesthesiology, The First Affiliated Hospital of Xi'an Jiaotong University, Xi'an, Shaanxi, China; ^2^Center for Translational Medicine, The First Affiliated Hospital of Xi'an Jiaotong University, Xi'an, Shaanxi, China

**Keywords:** inflammation-based markers, prognostic value, postoperative complications, short-term, colorectal tumor

## Abstract

**Background:**

Numerous inflammatory biomarkers have been identified to possess a positive prognostic value in relation to the clinical outcomes of patients with various cancers. Despite this, there is a paucity of comprehensive studies that compare the prognostic value of commonly used inflammatory parameters specifically within colorectal cancer (CRC) populations. These parameters include the peripheral blood neutrophil-to-lymphocyte ratio (NLR), platelet-to-lymphocyte ratio (PLR), systemic immune inflammation index (SII), albumin-to-globulin ratio (AGR), and prognostic nutritional index (PNI). Thus, the objective of this research was to conduct a thorough comparison of the predictive potential value of preoperative commonly used inflammatory response markers in CRC patients.

**Methods:**

This is a retrospective, single-center cohort analysis. We performed a secondary analysis of 392 individuals with CRC who fulfilled our inclusion criteria and were admitted to the First Affiliated Hospital of Xi'an Jiaotong University between June 2018 and August 2019. Initially, the clinical data including baseline demographics, laboratory indices, type of surgery, type of anesthesia, and postoperative complications were collected. Then, the prognostic efficacy and threshold values of preoperative inflammatory biomarkers were ascertained through the employment of receiver operating characteristic (ROC) curves. Finally, both univariate and multivariate analyses were conducted to discern the risk factors contributing to postoperative complications with CRC patients.

**Results:**

In the present study, 54 (13.78%) patients experienced surgical complications. According to ROC curve analysis, PNI possessed the strongest predictive ability for surgical complications (AUC = 0.706, 95% CI = 0.642–0.770; *p* = 0.001). Concurrently, the cut-off value of PNI was 48.78 based on the highest Youden index. Multivariate analysis demonstrated that PNI ≤ 48.78 (OR = 0.904, 95% CI = 0.844–0.967, *p* = 0.003) and laparotomy (OR = 1.863, 95% CI = 1.017–3.415, *p* = 0.044) emerged as independent risk factors for short-term postoperative complications. Lastly, the PNI ≤ 48.78 group exhibited an increased likelihood of requiring intraoperative blood transfusions and experienced extended duration of hospitalization.

**Conclusion:**

Preoperative PNI possesses superior ability and serves as an independent predictor of clinical complications following colorectal resection surgery. Multidisciplinary teams should focus on addressing patients' immunonutrition status before surgery.

## Introduction

In 2020, colorectal cancer (CRC) was identified as the second most prevalent malignancy and the fourth leading cause of cancer-related deaths in China (1). The mortality rate from CRC was estimated at 178.02 thousand across the nation (2), posing a significant threat to public health and exerting a substantial socioeconomic impact. Currently, surgical resection is upheld as the optimal treatment method for colorectal cancer, exhibiting promising early surgical results with a 5-year survival rate of 90% (3) and a 5-year tumor recurrence rate of 6.9% (4). Nonetheless, colorectal resections are linked with an eight-fold increase in the risk of negative outcomes in comparison to other abdominal procedures (5). In fact, up to 30% patients experience complications following colorectal surgery (6–9). This results in extended hospitalization periods, escalated costs, a reduction in the quality of life, and indirectly, inferior long-term outcomes due to the postponement of adjuvant treatment initiation. Consequently, there is a pressing necessity to pinpoint risk factors for complications following colorectal resections, in order to enhance the predictive capacity for the swift identification of severe postoperative complications and aid in the implement of personalized perioperative treatment measures.

Emerging empirical data indicates that the systemic inflammatory response has a crucial function in the progression of cancer (10, 11). Biomarkers of inflammation, derived from standard blood tests, can effectively mirror systemic inflammatory response that occurs within the host and hold predictive value that is independent of the TNM staging system (12, 13). At present, extensively investigated biomarkers including the peripheral blood neutrophil-to-lymphocyte ratio (NLR), platelet-to-lymphocyte ratio (PLR), systemic immune inflammation index (SII), albumin-to-globulin ratio (AGR), and prognostic nutritional index (PNI) are becoming increasingly useful in prognosis prediction. Previously, Pedrazzani et al. (14) compared 603 CRC patients and 5,270 healthy blood donors, and reported that there was an inverse association between a high NLR and a poorer survival rate. Krakowska et al. (15) involved 295 cases with advanced colorectal cancer who underwent palliative chemotherapy in the first line, and revealed that an elevated NLR and a high PLR were linked to a worse prognosis. Xie et al. (16) conducted a study with 240 patients suffering from metastatic colorectal cancer, and indicated that a high SII value significantly predicted a poor overall survival rate. Li et al. (17) found that a low AGR and a low PNI were significant predictors for worse overall survival and progression-free survival among CRC cases. Meanwhile, in CRC patients with metastatic disease, PNI level could also predict poor survival outcomes (18). Additionally, a recent systematic review was conducted with a focus on the correlation between PNI and the postoperative as well as survival outcomes among CRC patients, culminating in a similar conclusion (19). Unfortunately, this meta-analysis, which incorporated ten articles, revealed that only two articles explored the correlation between PNI values and complications following surgery.

As previously mentioned, the majority of the studies concentrated on the long-term prognosis, neglecting to consider short-term postoperative complications. It is crucial to note that inconsistent result has also been reported (20, 21). Furthermore, up until now, only a handful of studies have conducted a thorough comparison of the prognostic significance of these indicators on postoperative complications in patients with CRC, and the correlation between these indicators and intraoperative events remains unclear.

Hence, the aim of this research is to conduct a comprehensive comparison of the predictive potential value of preoperative commonly used inflammatory markers in relation to short-term postoperative complications following colorectal surgery. Additionally, the research seeks to investigate the correlation between these indicators and intraoperative events in patients with CRC.

## Materials and methods

### Study design and patients

This study is based on a secondary analysis of data collected from a preceding randomized clinical trial (NCT03086304) and received approval from the Ethics Committee of the First Affiliated Hospital of Xi'an Jiaotong University (Approval No. XJTU1AF2016LSL-035) (22). We performed a retrospective cohort study and prospectively gathered the perioperative data of patients undergoing elective colorectal surgery from the First Affiliated Hospital of Xi'an Jiaotong University over the period from June 2018 to August 2019. Written informed consent of all enrolled participants was obtained from the prior randomized clinical trial. This retrospective cohort study was performed in adherence to the provisions of the STrengthening the Reporting of OBservational studies in Epidemiology (STROBE) guidelines.

### Inclusion and exclusion criteria

The inclusion criteria were as follows: (1) patients who underwent elective colorectal surgery under general anesthesia; (2) patients over 18 years with an American Society of Anesthesiologist scores ranging between I and III. The exclusion criteria were as follows: (1) patients who underwent emergency surgery due to bowel obstruction, colonic perforation, or bleeding; (2) patients receiving neoadjuvant chemoradiation therapy; (3) patients who previously underwent intraoperative enterostomy; (4) patients with comorbid inflammatory diseases and active infection; and (5) pregnant or breast-feeding.

### Indicators and measurements

The following data were prospectively gathered from the hospital's electronic patient records: baseline characteristics, preoperative laboratory indices, intraoperative parameters, and postoperative indicators. Baseline characteristics comprised age, gender, body mass index (BMI), comorbidities, history of previous abdominal surgeries, and American Association of Anesthesiologists (ASA) score. Moreover, comorbidity scores based on the Age-Adjusted Charlson Comorbidity Index (ACCI) were calculated upon admission, and patients with ACCI equal to or above 4 were considered to have serious comorbidities (23).

Preoperative laboratory indices consisted of preoperative hemoglobin level (HGB), erythrocyte count, leukocyte count, platelet count, neutrophil percentage (%), lymphocyte percentage (%), and serum albumin and globulin levels within 2 weeks before surgery. Subsequently, inflammatory markers, including the NLR, PLR, SII, AGR, and PNI were derived from laboratory results of peripheral blood. Specifically, the NLR, PLR, and AGR were calculated by dividing the neutrophil count (10^9^/L) by the lymphocyte count (10^9^/L), the platelet count (10^9^/L) by the lymphocyte count (10^9^/L), and the serum albumin level (g/dl) by the globulin level (g/dl). SII was calculated as follows: platelet count (10^9^/L) × neutrophil count (10^9^/L)/lymphocyte count (10^9^/L). Lastly, PNI was calculated using the following formula: albumin (g/L) + 5 × total lymphocyte count (10^9^/L) (Table 1).

**Table 1:** Distribution of inflammation-related parameters in CRC patients.

**Parameters**	**Minimum value**	**Maximum value**	**Mean value**	**Standard deviation**
Platelet (10^9^/L)	82	740	246.28	87.546
Neutrophil percentage (10^9^/L)	0.88	19.85	3.802	1.776
Lymphocyte percentage (10^9^/L)	0.42	4.27	1.625	0.541
Albumin (g/dL)	26.500	51.5	39.100	4.349
Globulin (g/dL)	13.1	44.1	26.420	4.009
NLR	0.610	11.100	2.587	1.517
PLR	38.960	693.100	166.900	81.18
SII	100.120	5,385.410	658.000	558.046
AGR	0.810	2.940	1.510	0.260
PNI	30.850	65.000	47.224	5.433

NLR, neutrophil-to-lymphocyte ratio; PLR, platelet-to-lymphocyte ratio; SII, systemic immune inflammation index; AGR, albumin-to-globulin ratio; PNI, prognostic nutritional index.

Intraoperative parameters, such as the surgical modality, duration of operation, intraoperative drugs, and intake and output volumes (crystal, colloid, blood product infusion, bleeding, and urine volume) were collected. Postoperative indicators comprised tumor size, tumor staging, postoperative hospital stay, and postoperative complications. The pathological staging of these CRC patients was determined in accordance with the 7th edition of the International Union Against Cancer (UICC) TNM Classification. Postoperative complications, including postoperative bleeding, anastomotic leakage, surgical site infection, intra-abdominal abscess, intestinal obstruction, urinary retention, urinary tract infection, intestinal inflammation, pancreatic fistula, lymphorrhea, ascites, postoperative cardiovascular disease, pulmonary disease, and delirium were assessed utilizing the Clavien–Dindo classification (24). In this current research, the principal outcome evaluated was the occurrence of grade II or higher postoperative complications, as classified by the Clavien–Dindo system, within a 30-day post-surgery period. In instances where patients experienced multiple complications, the Clavien–Dindo grade was ascertained based on the most severe complication.

### Surgery and anesthesia procedures

All patients underwent elective colorectal resections under general anesthesia. The surgical intervention and postoperative therapeutic measures were entirely determined at the discretion of surgeons in accordance with current routine clinical practice. All operative procedures were conducted under a combination of intravenous and inhalational anesthesia, accompanied by endotracheal intubation. Prior to the induction of general anesthesia, 0.375% ropivacaine was administered for transverse abdominis plane block, with each injection consisting of 10 ml. Standardized general anesthesia was induced with 0.5 μg/kg sufentanil, 0.2 mg/kg etomidate, and 1 mg/kg rocuronium and then maintained with remifentanil, propofol, rocuronium, dexmedetomidine and sevoflurane. Following anesthesia induction, a peripheral arterial line was established to maintain the mean arterial blood pressure within 20% of preoperative levels during the intervention. During surgery, the dosage of anesthesia maintenance drugs was adjusted according to the bispectral index (between 40 and 60) and the circulatory parameters. Concurrently, an intraoperative fluid management program was formulated, taking into account the patient's fundamental vital signs and arterial blood gas parameters. After surgery, intravenous analgesia pumps were employed based on the analgesic requirement of the patients. The formula for the postoperative analgesia pump consisted of 100 μg of sufentanil and 10 mg of dexamethasone, diluted to 100 ml with normal saline.

### Statistical analysis

Continuous variables following a normal distribution were presented as mean ± SD and compared using *t*-test, whereas those following a non-normal distribution were expressed as median (interquartile range) and compared using the Mann–Whitney *U*-test. Data distribution was determined using the Kolmogorov–Smirnov test. Category variables were presented as numbers and percentages and compared using the Chi-square test or Fisher's exact test. The Receiver Operating Characteristic (ROC) curves were plotted, and the Youden index was estimated to define the optimal cut-off value. The Youden index was calculated using the following formula: sensitivity + specificity−1. In addition, independent variables with a *p*-value < 0.05 in the univariable logistic regression analysis, as well as those of clinical significance, were incorporated into the multivariable logistic regression model. These variables were assessed for multicollinearity using variance inflation factors (VIF), with a VIF of 5 or greater suggesting potential multicollinearity. Statistical analyses were conducted using SPSS software, version 23.0 (IBM SPSS Statistics for Windows, Version 23.0). A *p*-value of < 0.05 was considered statistically significant.

## Results

### Patients and classification

**Figure 1:**
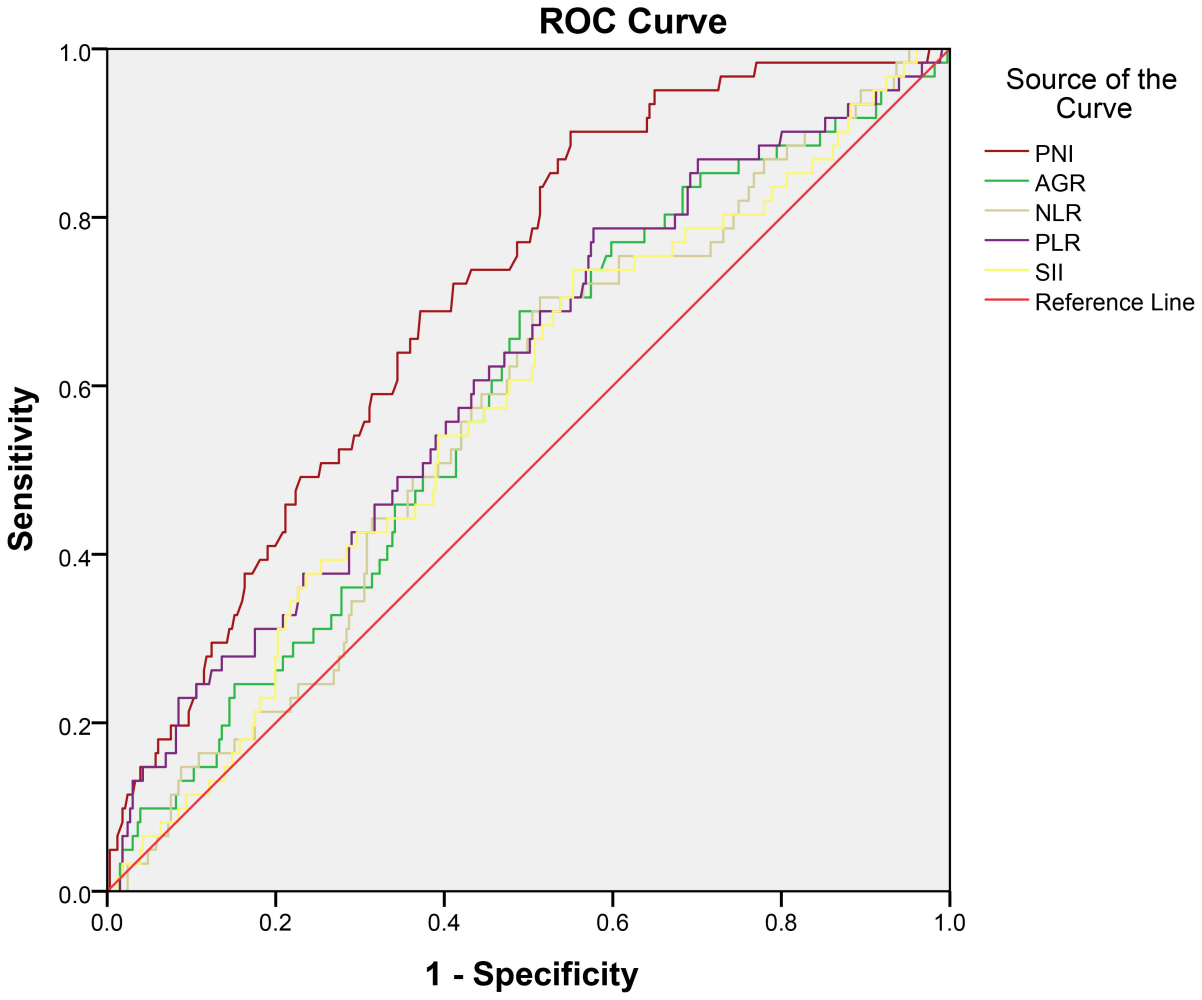
ROC curves for predicting postoperative complications in CRC patients.

**Figure 2:**
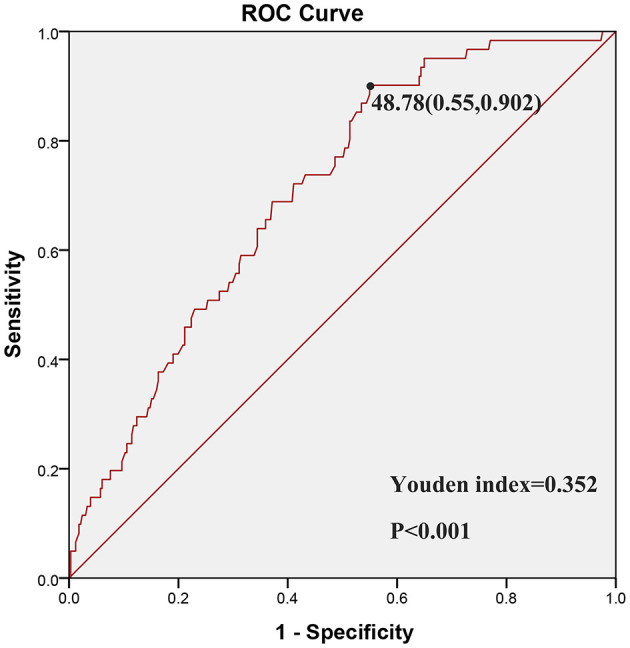
The cut-off value of PNI based on highest Youden index.

Between June 2018 and August 2019, a total of 413 patients underwent screening. Of these, 17 patients were excluded due to their receipt of preoperative neoadjuvant chemoradiation therapy. One patient was excluded owing to a histopathologically-confirmed diagnosis of lymphoma, one patient was excluded due to a histopathologically-confirmed diagnosis of intestinal tuberculosis, one patient was excluded due to a histopathologically-confirmed diagnosis of appendicitis, and one patient was excluded due to a histopathologically-confirmed diagnosis of Peutz-Jeghers syndrome. Out of the total, 392 patients, accounting for 94.92%, met the inclusion criteria and were subsequently included in the current study. There were 223 males (56.89%) and 169 females (43.11%), with a median age of 62 years (range 54–69 years). Among them, 54 (13.78%) patients experienced postoperative complications. No recorded instances of patient mortality within a 30-day postoperative period in our medical center. According to the ROC curve analysis, PNI exerted the strongest predictive ability for complications (AUC = 0.706, 95% CI = 0.642–0.770; *p* < 0.001; Figure 1 and Table 2). Meanwhile, the optimal cut-off value for PNI, determined by the maximum Youden index, was established at 48.78 (Figure 2). As a result, patients were stratified into two groups: those with a PNI ≤ 48.78 (*n* = 237, 60.46%) were classified as PNI-low, and those with a PNI >48.78 (*n* = 155, 41.5%) were classified as PNI-high.

**Table 2:** Predictive value of preoperative inflammatory markers for postoperative complications in CRC patients.

**Parameters**	**AUC**	**95% CI**	***P*-value**
NLR	0.568	0.494–0.643	0.089
PLR	0.612	0.535–0.689	0.005
SII	0.574	0.497–0.651	0.068
AGR	0.584	0.509–0.660	0.037
PNI	0.706	0.642–0.770	< 0.001

NLR, neutrophil-to-lymphocyte ratio; PLR, platelet-to-lymphocyte ratio; SII, systemic immune inflammation index; AGR, albumin-to-globulin ratio; PNI, prognostic nutritional index.

### Associations of PNI status with clinicopathological factors and postoperative outcomes

As expected, there was a significant correlation between the PNI status and specific clinicopathological factors, as detailed in Table 3. The group with low PNI was significantly associated with lower BMI (22.55 ± 3.06 vs. 23.18 ± 2.91, *p* = 0.043), higher ASA grades (14.35 vs. 3.87%, *p* = 0.001), severe preoperative anemia (112.39 ± 21.29 vs. 135.28 ± 22.19, *p* = 0.000), and larger tumor sizes (4.90 ± 1.87 vs. 4.32 ± 1.91, *p* = 0.003) in comparison to the group with high PNI. On the other hand, the remaining demographic characteristics, prevalence of comorbidities, and tumor characteristics were no comparable between the two groups. As presented in Table 4, the PNI-low group had a higher proportion of cases who underwent laparotomy surgery and colectomy than those in the PNI-high group (36.29 vs. 24.52%, *p* = 0.014; 64.98 vs. 52.26%, *p* = 0.012). During the operation, patients in the PNI-low group received a lower amount of colloidal fluids but a higher amount of blood products, including red blood cells and plasma, than cases in the PNI-high group (796.62 ± 337.35 vs. 883.87 ± 300.54, *p* = 0.008; 15.61 vs. 6.45%, *p* = 0.006; 12.24 vs. 1.94%, *p* = 0.000). Furthermore, when compared to the group with low PNI, the group with high PNI demonstrated a significantly reduced duration of hospital stay and a lower frequency of postoperative complications (18.35 ± 5.89 vs. 16.63 ± 4.50, *p* = 0.001; 20.25 vs. 3.87%, *p* = 0.000).

**Table 3:** Demographic and clinical characteristics of patients after colorectal surgery.

**Variables**	**Total (*n* = 392)**	**PNI-low (*n* = 237)**	**PNI-high (*n* = 155)**	***P*-value**
Age (years)	62 (54.0–69.0)	62 (54.0–69.0)	61 (53.0–68.0)	0.137
Gender (F/M)	169/223	110/127	59/96	0.103
BMI (kg/m^2^)	22.80 ± 3.01	22.55 ± 3.06	23.18 ± 2.91	0.043
Smoking, *n* (%)	109 (27.81)	60 (25.32)	49 (31.61)	0.174
**ASA**, ***n*** **(%)**				0.001
II	352 (82.40)	203 (85.65)	149 (96.13)	
III	40 (10.20)	34 (14.35)	6 (3.87)	
ACCI	2 (1–3)	2 (2–3)	2 (2–3)	0.590
Hypertension, *n* (%)	85 (21.68)	53 (22.36)	32 (20.65)	0.687
Coronary heart disease, *n* (%)	16 (4.08)	13 (5.49)	3 (1.94)	0.082
History of abdominal surgery, *n* (%)	26 (6.63)	18 (25.32)	8 (5.16)	0.344
Preoperative hemoglobin (g/L)	121.44 ± 24.35	112.39 ± 21.29	135.28 ± 22.19	0.000
Tumor size (cm)	4.67 ± 1.90	4.90 ± 1.87	4.32± 1.91	0.003
**Depth of infiltration**, ***n*** **(%)**				0.218
T0	14 (3.57)	9 (3.80)	5 (3.23)	
Tis	7 (1.79)	4 (1.69)	3 (1.94)	
T1	21 (5.36)	10 (4.22)	11 (7.10)	
T2	60 (15.31)	31 (13.08)	29 (18.71)	
T3	128 (32.65)	74 (31.22)	54 (34.84)	
T4	162 (41.33)	109 (45.99)	53 (34.19)	
**Lymph node metastasis**, ***n*** **(%)**				0.522
N0	253 (64.54)	150 (63.29)	103 (66.45)	
N1	139 (35.46)	87 (36.71)	52 (33.55)	
**Distant metastasis**, ***n*** **(%)**				0.467
No	373 (95.15)	224 (94.51)	149 (96.13)	
Yes	19 (4.85)	13 (5.49)	6 (3.87)	
**TNM stage**, ***n*** **(%)**				0.511
0	8 (2.04)	5 (2.19)	3 (2.00)	
I	70 (17.86)	36 (15.79)	34 (22.67)	
II	154 (39.29)	97 (42.54)	57 (38.00)	
III	127 (32.40)	77 (33.77)	50 (33.33)	
IV	19 (4.85)	13 (5.70)	6 (4.00)	

BMI, body mass index; ASA, American Association of Anesthesiologists score; ACCI, Age-Adjusted Charlson Comorbidity Index.

**Table 4:** Perioperative data of patients after colorectal surgery.

**Variables**	**Total (*n* = 392)**	**PNI-low (*n* = 237)**	**PNI-high (*n* = 155)**	***P*-value**
**Surgical method**				0.014
Laparoscopic, *n* (%)	268 (68.37)	151 (63.71)	117 (75.48)	
Laparotomy, *n* (%)	124 (31.63)	86 (36.29)	38 (24.52)	
**Colorectal resection**, ***n*** **(%)**				0.012
Colectomy, *n* (%)	235 (59.95)	154 (64.98)	81 (52.26)	
Proctectomy, *n* (%)	157 (40.05)	83 (35.02)	74 (47.74)	
Operation time (h)		3.50 ± 1.22	3.41± 0.98	0.396
Any vasopressor used, *n* (%)	64 (16.33)	43 (18.14)	21 (13.55)	0.264
**General anesthetic**				
Propofol (mg)	917.834 ± 380.685	935.09 ± 406.52	891.45 ± 336.95	0.268
Etomidate (mg)	13.383 ± 3.276	13.19 ± 3.33	13.68 ± 3.19	0.150
Sevoflurane (ml)	38.207 ± 15.029	38.33 ± 15.17	38.02 ± 14.85	0.842
Dexmedetomidine (μg)	80.770 ± 38.096	81.90 ± 40.45	79.05 ± 34.24	0.469
Remifentanil (μg)	1,786.982 ± 660.227	1,797.62 ± 698.92	1,770.71 ± 598.07	0.694
Sufentanil (μg)	129.972 ± 10.898	129.97 ± 9.68	131.27 ± 5.15	0.127
Cisatracurium bromide (mg)	24.645 ± 11.028	24.30 ± 10.85	25.18 ± 11.31	0.438
Rocuronium bromide (mg)	54.313 ± 21.231	54.32 ± 20.71	54.31 ± 22.07	0.998
**Total infusion**				
Intraoperative crystalloid fluid (ml)	1,786.276 ± 560.678	1,815.27 ± 555.51	1,741.94 ± 567.42	0.206
Intraoperative colloidal fluid (ml)	831.122 ± 325.711	796.62 ± 337.35	883.87 ± 300.54	0.008
Intraoperative red blood cell, *n* (%)	47 (11.99)	37 (15.61)	10 (6.45)	0.006
Intraoperative plasma, *n* (%)	33 (8.42)	29 (12.24)	3 (1.94)	0.000
Intraoperative blood loss (ml)	136.798 ± 112.944	142.76 ± 112.89	127.68 ± 112.79	0.196
Urine volume (ml)	776.429 ± 516.486	760.42 ± 512.87	800.90 ± 522.68	0.449
Postoperative analgesia, *n* (%)	384 (97.96)	231 (97.47)	153 (98.71)	0.628
Length of stay in hospital (day)	17.666 ± 5.442	18.35 ± 5.89	16.63 ± 4.50	0.001
Postoperative complications, *n* (%)	54 (13.78)	48 (20.25)	6 (3.87)	0.000

### Univariate and multivariate analyses of risk factors for postoperative complications

In this study, 54 patients developed Clavien–Dindo grade II or higher postoperative complications. Postoperative complications included pneumonia in two cases, bacteremia in two cases, anastomotic leakage in 15 cases, wound infection in 12 cases, arrhythmia in two cases, ileus in 12 cases, deep venous thrombosis (DVT) in one case, acute renal dysfunction in one case, delirium in one case, pancreatic fistula in three cases, gastrointestinal bleeding in two cases, acute left heart failure in one case, and acute cerebral infarction in two cases (Table 5). Among these, six patients experienced two types of complications, whilst three patients experienced three types of complications. Univariate analysis demonstrated that a previous history of abdominal surgery [odds ratio (OR) = 2.625, 95% confidence interval (CI): 1.086–6.342, *p* = 0.032], preoperative anemia (OR = 2.528, 95% CI = 1.451–4.407, *p* = 0.001), laparotomy (OR = 1.916, 95% CI = 1.097–3.347, *p* = 0.022), recipient of blood products (OR = 2.088, 95% CI = 1.051–4.151, *p* = 0.036) and PNI ≤ 48.78 (OR = 0.133, 95% CI = 0.056–0.318, *p* = 0.000) were significantly correlated with the incidence of postoperative complications (Table 6). Based on clinical evaluation, the history of abdominal surgery was determined to have no significant impact on short-term postoperative complications. Subsequently, the presence of multicollinearity between variables was assessed using tolerance values and VIF. The multicollinearity test revealed that the maximum VIF was 1.399, with a mean VIF of 1.238, and the minimum tolerance value was 0.715, confirming the absence of multicollinearity among covariates (Table 7). Consequently, four significant variables—preoperative anemia, surgical method, receipt of blood products, and PNI—were included in the multivariate analysis to identify independent risk factors for postoperative complications following CRC surgery. Ultimately, a PNI of 48.78 or lower (OR = 0.904, 95% CI = 0.844–0.967, *p* = 0.003) and laparotomy (OR = 1.863, 95% CI = 1.017–3.415, *p* = 0.044) were identified as independent risk factors for short-term postoperative complications.

**Table 5:** Postoperative complications after surgery in patients with CRC.

**Complications**	**Number**	**Total**
**Grade II**		48
Pneumonia	2	
Bacteremia	2	
Anastomotic leakage	15	
Wound infection	12	
Arrthymia	2	
Ileus	12	
DVT	1	
Acute renal dysfunction	1	
Delirium	1	
**Grade III**		5
Pancreatic fistula	3	
Gastrointestinal bleeding	2	
**Grade IV**		3
Acute left heart failure	1	
Acute cerebral infarction	2	

**Table 6:** Univariate and multivariate analysis of risk factors associated with postoperative complications of patients after colorectal surgery.

**Variables**	**Univariable analysis**		**Multivariate analysis**	
	**OR (95% CI)**	***P*** **-value**	**OR (95% CI)**	***P*** **-value**
**Age (** ***n*** **, %)**				
< 60 years	1 (Reference)			
≥60 years	0.993 (0.969–1.016)	0.534		
**Gender (** ***n*** **, %)**				
Female	1 (Reference)			
Male	0.875 (0.505–1.514)	0.632		
**BMI (** ***n*** **, %)**				
≤ 18.5 kg/m^2^	1 (Reference)			
>18.5 kg/m^2^	1.779 (0.767–4.125)	0.180		
**Smoking (** ***n*** **, %)**				
No	1 (Reference)			
Yes	0.765 (0.592–2.040)	0.765		
**ASA (** ***n*** **, %)**				
II	1 (Reference)			
III	0.671 (0.229–1.967)	0.467		
**ACCI**				
< 4	1 (Reference)			
≥4	0.434 (0.100–1.884)	0.265		
**Hypertension**, ***n*** **(%)**				
No	1 (Reference)			
Yes	0.866 (0.437–1.713)	0.678		
**Coronary heart disease**, ***n*** **(%)**				
No	1 (Reference)			
Yes	0.351 (0.046–2.708)	0.315		
**History of abdominal surgery**, ***n*** **(%)**				
No	1 (Reference)			
Yes	2.625 (1.086–6.342)	0.032		
**Preoperative anemia, (** ***n*** **, %)**				
No	1 (Reference)		1 (Reference)	
Yes	2.528 (1.451–4.407)	**0.001**	1.266 (0.624 −2.567)	0.514
**Colorectal resection**, ***n*** **(%)**				
Colectomy	1 (Reference)			
Proctectomy	0.819 (0.465–1.443)	0.490		
**Tumor size**, ***n*** **(%)**				
≤ 5 cm	1 (Reference)			
>5 cm	1.774 (0.643–2.144)	0.602		
**Depth of infiltration**, ***n*** **(%)**				
≤ T2	1 (Reference)			
>T2	1.963 (0.956–4.030)	0.066		
**Lymph node metastasis**, ***n*** **(%)**				
N0	1 (Reference)			
N1	1.032 (0.584–1.823)	0.914		
**Distant metastasis**, ***n*** **(%)**				
No	1 (Reference)			
Yes	2.022 (0.700–5.835)	0.193		
**TNM stage**, ***n*** **(%)**				
≤ II stage	1 (Reference)			
>II stage	1.024 (0.582–1.799)	0.936		
**Surgical method**				
Laparoscopic, *n* (%)	1 (Reference)		1 (Reference)	
Laparotomy, *n* (%)	1.916 (1.097–3.347)	**0.022**	1.863 (1.017–3.415)	0.044
**Operation time (** ***n*** **, %)**				
≤ 3 h	1 (Reference)			
>3 h	1.268 (0.719–2.233)	0.412		
**Blood loss (ml)**				
Blood loss ≤ 200 ml	1 (Reference)			
Blood loss >200 ml	1.719 (0.789–3.747)	0.173		
**Blood products**, ***n*** **(%)**				
No	1 (Reference)		1 (Reference)	
Yes	2.088 (1.051–4.151)	**0.036**	1.031 (0.449–2.368)	0.943
**PNI (** ***n*** **, %)**				
≤ 48.78	1 (Reference)		1 (Reference)	
>48.78	0.133 (0.056–0.318)	**0.000**	0.904 (0.844–0.967)	0.003

BMI, body mass index; ASA, American Association of Anesthesiologists score; ACCI, Age-Adjusted Charlson Comorbidity Index; PNI, prognostic nutritional index. Significant *P* values were expressed bold characters.

**Table 7:** Multicollinearity test to examine the relationship between explanatory variables.

**Variables**	**VIF**	**1/VIF**
Preoperative anemia	1.399	0.715
Blood products	1.220	0.820
Surgical method	1.033	0.968
PNI	1.301	0.769
Mean VIF	1.238	

## Discussion

The present retrospective study scrutinized the clinical significance of preoperative inflammatory markers in a sample of 392 cases with CRC who underwent elective colorectal resection. The findings revealed that the PNI possessed the greatest predictive value for postoperative complications following colorectal resections (AUC = 0.706, 95% CI = 0.642–0.770; *p* = 0.000) when compared to other common preoperative inflammatory markers including NLR, PLR, SII, and AGR. Concurrently, both univariate and multivariate logistic regression analyses indicated that a preoperative PNI of 48.78 or lower (OR = 0.904, 95% CI = 0.844–0.967, *p* = 0.003) could significantly predict complications following surgery. Therefore, preoperative PNI could serve as a strong and independent predictor of complications following colorectal resection surgery among CRC patients.

In our medical center, we observed a 13.78% incidence rate of postoperative complications, a figure that aligns with the findings reported by Cao et al. (25). However, this figure was marginally lower than the 10% reported in previous studies carried out by Portale et al. (20) and Tokunaga et al. (26). These inconsistencies may be attributed to the following: firstly, study participants were ethnically diverse. Secondly, our institution, a comprehensive tertiary hospital located in a large urban area, had a large volume of CRC patients. The specialized skills of clinicians and effective perioperative management could have contributed to superior clinical outcomes. As is well-established, systemic inflammatory responses play a decisive role in various postoperative complications. Among the common preoperative inflammatory markers, PNI was shown to have the lead performance in predicting complications in CRC patients (AUC = 0.706, 95% CI = 0.642–0.770; *p* = 0.001). The research by Imai et al. (27) similarly found that PNI had superior ability in predicting overall survival and recurrence-free survival for patients who underwent curative liver resection. Based on the maximal Youden index, patients were stratified into the PNI-low group (PNI ≤ 48.78) and the PNI-high group (PNI > 48.78). Notably, the incidence of postoperative complications was 20.25% in the PNI-low group, compared to 3.87% in the PNI-high group. In other words, those in the low PNI group exhibited a prevalence of complications nearly five times higher than those in the high PNI group. This strong association may have been influenced by the presence of other disorders, surgical factors, and the relatively small sample size.

The PNI, derived from serum albumin levels and total lymphocyte count, serves as an innovative systemic inflammatory marker, providing insight into the nutritional and immunological status of cancer patients (28). Prior research has indicated that the PNI holds significant prognostic value across a range of cancer types, including colorectal cancer (19), esophageal cancer (29), gastric cancer (30), pancreatic cancer (31), hepatocellular carcinoma (32), breast cancer (33), ovarian cancer (34), and lung cancer (35). Meanwhile, existing evidence has established that preoperative poor immunonutrition promotes the development of postoperative short-term complications (36, 37). Indeed, Yu et al. (38) provided evidence that the PNI could predict postoperative pulmonary complications in patients who underwent radical cystectomy. Various studies have proposed an association between PNI and postoperative acute kidney injury in patients who underwent coronary artery bypass grafting (39), living donor liver transplantation (40) and colorectal cancer surgery (41), respectively. Recently, Wei et al. (42) indicated that preoperative PNI associated with anastomotic leakage following colorectal surgery. Xie et al. (43) found a significant correlation between a low PNI status and increased postoperative complications in CRC patients who underwent surgical procedures. These conclusions supported the research results of the present study, indicating the association between preoperative PNI and short-term postoperative complications following colorectal surgery. This investigation further conducted a comprehensive comparison of the common preoperative inflammatory markers including the NLR, PLR, SII, AGR, and PNI on postoperative complications in CRC patients, and found that PNI possesses the most significant predictive value for postoperative complications following colorectal resections. We have a clinical significance to our study since it is the first to suggest that PNI is an optimal parameter in predicting short-term postoperative complications following colorectal surgery and thus clinicians should pay more attention on the preoperative PNI. The aforementioned study (20) presented varied results regarding the correlation between preoperative PNI and the incidence of postoperative complications in patients with rectal cancer undergoing laparoscopic resection. However, this study did not exclude patients who were administered neoadjuvant chemotherapy, a treatment known to be associated with hematological and gastrointestinal reactions (44). These reactions could potentially influence the preoperative PNI. Therefore, in our research, we have elected to exclude patients who underwent neoadjuvant chemotherapy. Coincidentally, Cao et al. (25) adopted the same exclusion criteria.

Our research findings indicate a significant correlation between a PNI of 48.78 or lower and the occurrence of postoperative complications, as determined by the ROC curve analysis. This is in line with previous studies that have employed varying PNI cut-off values. For instance, Cao et al. (25), Tokunaga et al. (26), and Xie et al. (43) used ROC curve analysis to establish PNI cut-off values of 45, 45.5, and 44.65, respectively. In another study, Mohri et al. (45) adopted the median PNI value of 45 as their cut-off point. Sim et al. (41) on the other hand, segmented their study participants into four groups based on PNI quartiles. Nozoe et al. (46) established a cutoff value of 49.7 derived from the mean value of the PNI. A subsequent study by Li et al. (17) identified an optimal PNI cutoff value of 48.65 based on the ROC curve analysis, which is consistent with our finding. The observed discrepancies may be attributed to variations in participant demographics across different geographical regions with differing trophic statuses.

Despite these findings, the potential mechanisms underpinning the correlation between PNI status and clinical outcomes remain inadequately understood (47). As is well-known, albumin levels reflect the nutritional status of patients. Under conditions of high inflammation, serum albumin may be reduced due to both liver reordering of protein synthesis and redistribution of albumin both outside and inside blood vessels (48). As a result of hypoalbuminemia, there is a delay in the healing of tissues, a reduction in collagen synthesis, and an impaired immune response (49). Meanwhile, lymphocytes play an essential role in regulating immune system pathways (50). A reduction in lymphocyte count results in immunosuppression and cytotoxic destruction. Possibility, the hypoalbuminemia and lymphopenia may mutually interact, further contributing to an increased susceptibility to unfavorable microenvironment for surgical patients (51). Therefore, preoperative PNI, combined the parameters of nutrition and immune, comprehensively evaluates the preoperative immunonutrition status of patients. A low-PNI score implies a compromised general condition, which renders patients less capable of withstanding the perioperative strikes and subsequent complications.

Furthermore, the present study revealed a significant inverse correlation between PNI status and ASA grade (*p* = 0.001), tumor size (*p* = 0.003), and laparotomy (*p* = 0.014). Conversely, a positive correlation was observed between PNI status and BMI (*p* = 0.043) as well as preoperative hemoglobin levels (*p* = 0.000). These findings substantiate the assertion that patients with lower preoperative PNI values generally present in a poorer condition prior to surgery. The findings of the current study align with those of Xie et al. (43), demonstrating that lower PNI was significantly associated with lower BMI and larger tumor (both *p* < 0.001). Notably, our study found that the PNI status was inversely correlated with intraoperative red blood cell transfusions (*p* = 0.006) and intraoperative plasma transfusion volumes (*p* = 0.000). The prior study conducted by Sim et al. (41) examined this association and discovered no statistically significant disparities among the four groups, as divided by the quartile of PNI. Nevertheless, a rise in PNI value corresponded to a decline in the percentage of patients requiring RBC transfusion (5.8% vs. 2.3% vs. 2.0% vs. 1.1%). This implies a potential association between PNI status and the likelihood of intraoperative blood transfusion.

Increasingly, hospital stays are being monitored for the length of time they last. The length of patient stay is considered a key indicator of the postoperative recovery rate. To date, the relationship between PNI and hospital stay has only been studied in a few previous studies (20, 41). Herein, the preoperative PNI value was negatively correlated with hospital stay (18.35 ± 5.89 in the PNI-low group vs. 16.63 ± 4.50 in the PNI-high group, *p* = 0.001). While the duration of hospitalization might be attributed to multiple factors, among which the poor immunonutrition status of patients should be recognized as an important contributor. Earlier studies evinced a significantly negative correlation between PNI values and the TNM stage (25, 45). However, in the current study, no significant correlation was observed between PNI and the TNM stage. Notwithstanding, our results exposed that those cases in the PNI-low group were more prone to developing advanced tumor infiltration depth (45.99 vs. 34.19% in the T4 stage), positive lymph node metastasis (36.71 vs. 33.55%), and distant metastasis (5.49 vs. 3.87%). It is worthwhile to emphasize that lower PNI values may potentially exacerbate malignant behaviors of tumor cells, leading to poorer clinical outcomes for patients with CRC. The absence of a statistically significant difference may potentially be attributed to the variance in the cut-off value of PNI. In our study, the determination of the PNI cut-off value was based on postoperative complications as the endpoint. While the referenced studies (25, 45) established the PNI cut-off value with survival rate as the endpoint.

The strengths of our study were the comprehensive comparisons of common preoperative inflammatory markers, namely NLR, PLR, SII, AGR, and PNI, and the analysis of intraoperative factors. Among these inflammatory markers, PNI exhibits a strong prediction capability in the prediction of short-term postoperative complications following colorectal surgery and is an optimal parameter for clinicians to identity the high risk of postoperative complications. In addition, there is certain relation between the PNI level and the risk of intraoperative blood transfusion. Therefore, before the operation, surgeons should be aware that enteral nutrition may be needed to enhance the ability of withstanding the perioperative strikes for patients with low PNI values. Notably, preoperative visit is an important part of the clinical work for anesthetists. Qualified anesthetists should be able to predict intraoperative and postoperative events based on the preoperative status of patients and thus formulate an ideal anesthesia plan to optimize intraoperative management. After the operation, it is necessary to strengthen the postoperative nursing for the high-risk cases. To this end, multidisciplinary teams should take steps to recognize and minimize the possible impact of the patient's immunonutrition status.

However, it is crucial to recognize the inherent limitations of this study. Primarily, the research was conducted retrospectively within a single center, resulting in an incomplete adjustment for potential confounders. Additionally, there were no established guidelines for nutritional support in surgical patients with a low PNI. Therefore, large-scale randomized trials investigating these areas are necessary for additional validation. Our research team intends to explore nutritional support's role in reducing intraoperative and postoperative complications for low-PNI surgical patients.

## Conclusion

In conclusion, preoperative PNI possesses superior ability and serves as an independent predictor of clinical complications following colorectal resection surgery. This tool could assist in identifying patients who are at an elevated risk of requiring intraoperative blood transfusions, experiencing postoperative complications, enduring extended hospital stays, and having a reduced rate of postoperative recovery. It is incumbent upon multidisciplinary teams, comprising surgeons, anesthetists, and nursing staff, to intensify their efforts to identify and mitigate the potential effects of the patient's immunonutrition status before surgery.

## Data Availability

The original contributions presented in the study are included in the article/supplementary material, further inquiries can be directed to the corresponding authors.
